# Supplementary material for: Moderately Low Effectiveness of the Influenza Quadrivalent Vaccine: Potential Mismatch between Circulating Strains and Vaccine Strains
Source: Vaccines (Basel). 2023 May 31;11(6):1050. doi: 10.3390/vaccines11061050 (PMC10304586; doi:10.3390/vaccines11061050)
Supplement: Supplementary file 1 [file vaccines-11-01050-s001.zip › vaccines-2396382-supplementary.pdf]

Supplementary Material

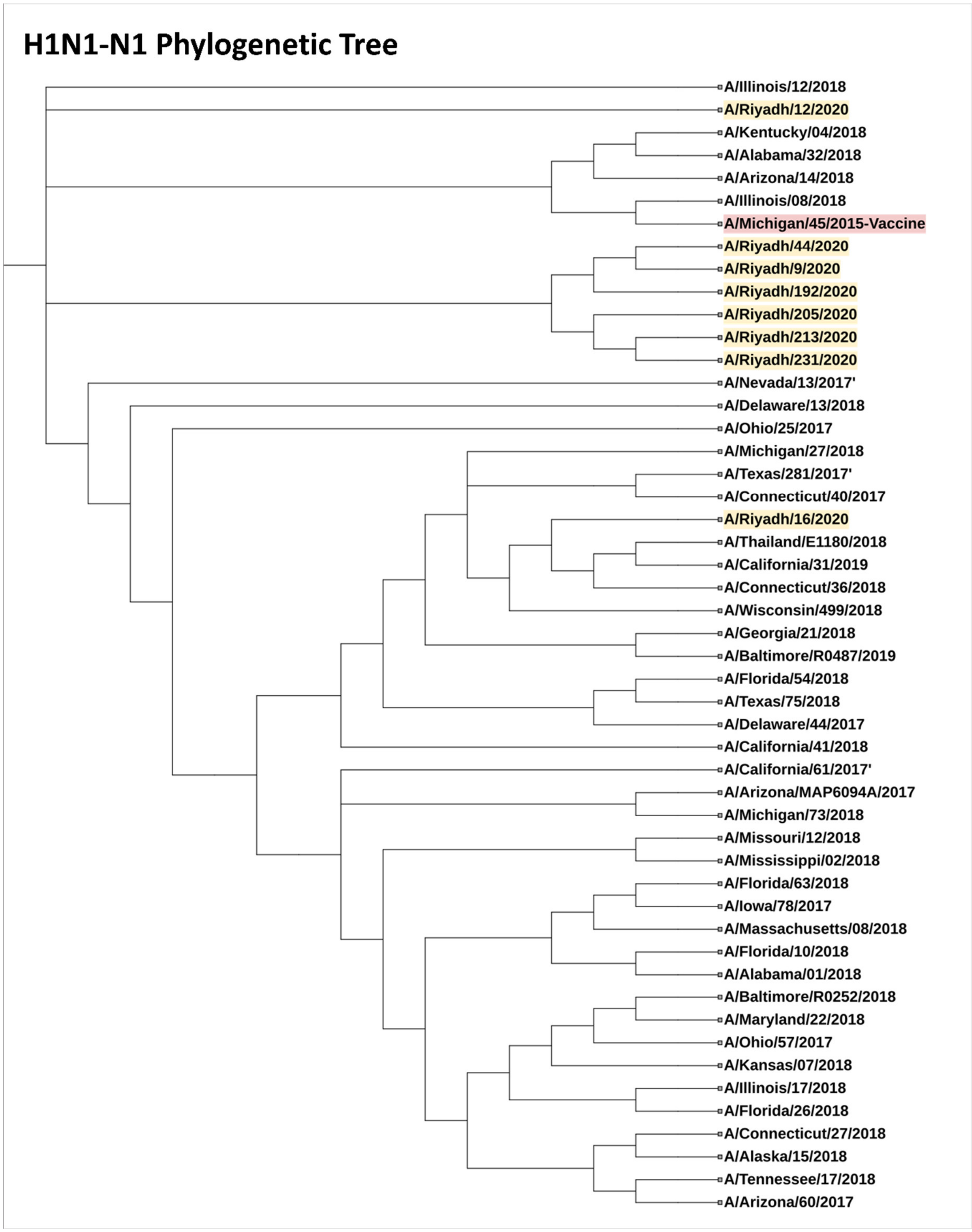

Figure S1: Phylogenetic tree of H1N1-Neuraminidase (NA) gene showing the genetic diversity and evolutionary relationships among the analyzed samples.

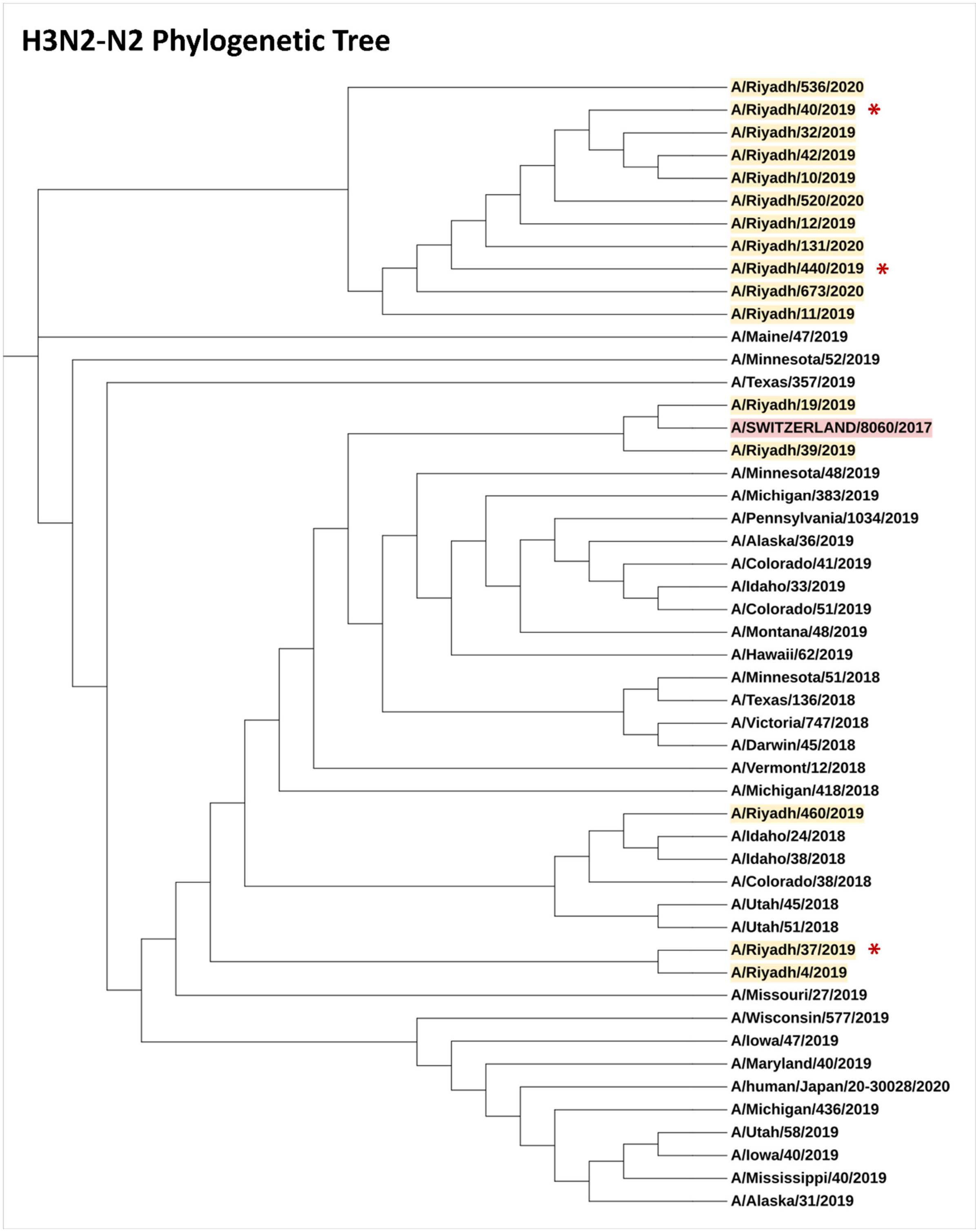

Figure S2: Phylogenetic tree of H3N2-Neuraminidase (NA) segment showing the genetic diversity and evolutionary relationships among the analyzed samples. The starred strains (*) are for vaccinated patients.
